# One crisis, diverse impacts—Tissue-specificity of folate deficiency-induced circulation defects in zebrafish larvae

**DOI:** 10.1371/journal.pone.0188585

**Published:** 2017-11-27

**Authors:** Hung-Chi Tu, Gang-Hui Lee, Tsun-Hsien Hsiao, Tseng-Ting Kao, Tzu-Ya Wang, Jen-Ning Tsai, Tzu-Fun Fu

**Affiliations:** 1 The Institute of Basic Medical Sciences, National Cheng Kung University, College of Medicine, Tainan, Taiwan; 2 Department of Medical Laboratory Science and Biotechnology, National Cheng Kung University, College of Medicine, Tainan, Taiwan; 3 Department of Medical Laboratory and Biotechnology, Chung Shan Medical University, Taichung, Taiwan; 4 Department of Clinical Laboratory, Chung Shan Medical University Hospital, Taichung, Taiwan; University of Maryland Center for Environmental Science, UNITED STATES

## Abstract

Folate (vitamin B9) is an essential nutrient required for cell survival, proliferation, differentiation and therefore embryogenesis. Folate deficiency has been associated with many diseases, including congenital heart diseases and megaloblastic anemia, yet the mechanisms underlying these remains elusive. Here, we examine the impact of folate deficiency on the development of the circulation system using a zebrafish transgenic line which displays inducible folate deficiency. Impaired hematopoiesis includes decreased hemoglobin levels, decreased erythrocyte number, increased erythrocyte size and aberrant *c-myb* expression pattern were observed in folate deficient embryos. Cardiac defects, including smaller chamber size, aberrant cardiac function and *cmlc2* expression pattern, were also apparent in folate deficient embryos. Characterization of intracellular folate content in folate deficiency revealed a differential fluctuation among the different folate derivatives that carry a single carbon group at different oxidation levels. Rescue attempts by folic acid and nucleotides resulted in differential responses among affected tissues, suggesting that different pathomechanisms are involved in folate deficiency-induced anomalies in a tissue-specific manner. The results of the current study provide an explanation for the inconsistent outcome observed clinically in patients suffering from folate deficiency and/or receiving folate supplementation. This study also supports the use of this model for further research on the defective cardiogenesis and hematopoiesis caused by folate deficiency.

## Introduction

Folate deficiency (FD) is one of the most common problems encountered clinically in malnutrition. FD has profound impact on embryogenesis and has been linked to several prevalent diseases, including anemia, neural tube defects and congenital heart disease, which is the most common congenital birth defect leading to fetal death [1, 2]. However, the underlying mechanism causing these diseases remains elusive. The benefits of folate supplementation on preventing FD and the associated diseases have been well-established. However, detrimental effects of folate supplementation, including increased risk of infant bronchiolitis, childhood asthma, cancer and chemoresistance have also been reported [3–14]. Lower circulating folate has been associated with reduced susceptibility to congenital heart disease in a subpopulation with specific gene variants [4]. These conflicting results on the efficacy of folate supplementation have raised concern and debate on the policy of mandatory folate fortification of food and maternal folic acid supplementation, a prevalent world-wide practice. Information on adequate dietary intake of essential nutrients, including folate, has been established to provide guidance for healthy individuals. However, these recommendations may not apply to individuals with special physiological conditions, such as pregnancy, disturbed immunity, chronic diseases, vulnerable gene variants and cancers [15, 16]. Therefore, understanding how folate contributes to normal physiological functions and how FD affects disease pathogenesis becomes imperative.

**Fig 1:**
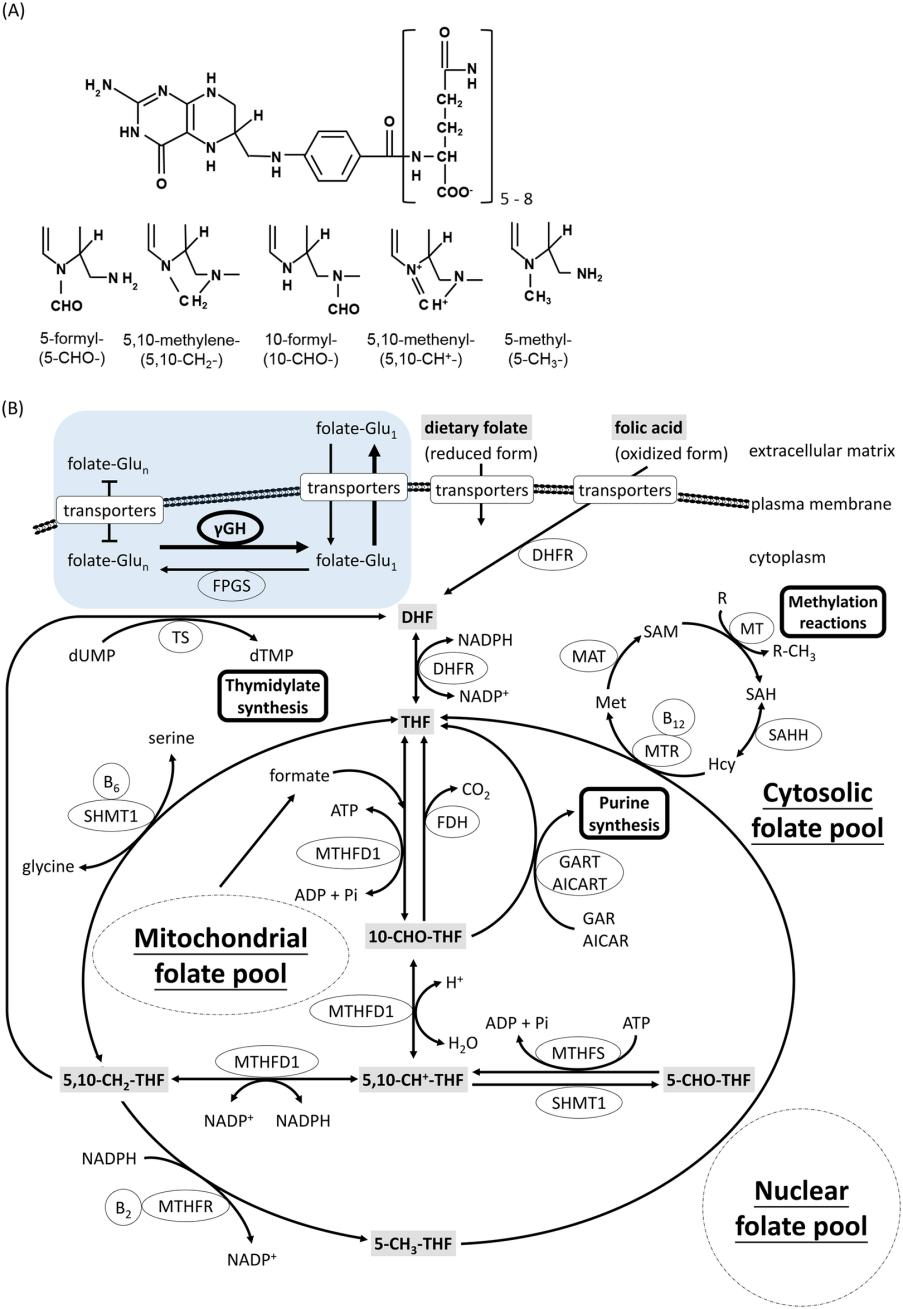
Folate and folate-mediated one-carbon metabolism. (A) Folate is comprised of a pteridine ring, p-aminobenzoic acid and glutamyl moieties with 5 to 8 glutamate residues attached in γ-linkage. The one-carbon units are attached to the N5- and/or N10-position of the pteridine ring at the oxidation levels of formate, formaldehyde and methanol. (B) The one-carbon units carried by reduced folate are involved in cytosolic, mitochondrial and nuclear folate pools and used for the biosynthesis of purines, thymidylate, amino acids and S-adenosylmethionine (SAM). Over-expressed γ-glutamylhydrolase (γGH) facilitates intracellular folate exportation by converting polyglutamylfolates (folate-Glu_n_) to monoglutamylfolates (folate-Glu_1_), leading to intracellular folate deficiency (thickened circle and arrows in shadowed box). Enzyme abbreviations: DHFR, dihydrofolate reductase; MTHFD, methylenetetrahydrofolate dehydrogenase; FDH, 10-formyltetrahydrofolate dehydrogenase; GART, glycinamide ribonucleotide transformylase; AICART, aminoimidazolecarboxamide ribonucleotide transformylase; MTHFS, 5,10-methenyltetrahydrofolate synthetase; SHMT, Serine hydroxymethyltransferase; MTHFR, methylenetetrahydrofolate reductase; TS, thymidylate synthase; MTR, 5-methyltetrahydrofolate-homocysteine methyltransferase; MAT, methionine adenosyl transferase; MT, methyltransferase; SAHH, S-adenosylhomocysteine hydrolase.

Folate, also known as vitamin B9, is essential for cell survival, proliferation and differentiation, hence crucial for embryogenesis. Folate is comprised of a pteridine ring, a *p*-aminobenzoic acid, a polyglutamyl moiety and carries one-carbon units on the N5/N10 of the pteridine ring (Fig 1A). The biologically active forms of folate are fully reduced tetrahydrofolate (THF). Natural folates are a mixture of THF carrying one-carbon units at the oxidation levels of formate, formaldehyde and methanol, forming various folate adducts with different activities. In cells, folate exists as polyglutamates with 5–8 glutamate residues attached to the γ–carboxyl of the first glutamate [17]. This polyglutamate tail functions to retain folate molecules inside the cell and to significantly increase the affinity to folate enzymes [18]. Folate participates in the biosynthesis of purine, pyrimidine, amino acids and neurotransmitters through folate-mediated one-carbon metabolism (Fig 1B). The formyl group on N_10_ of 10-CHO-THF is incorporated into purines during *de novo* biosynthesis and the methylene group of 5,10-methylenetetrahydrofolate (5,10-CH_2_-THF) is required for dTMP formation. In addition, the methyl group carried on 5-methyltetrahydrofolate (5-CH_3_-THF) is the one-carbon unit transfered for the formation of S-adenosylmethione (SAM), the methyl donor for most intracellular methylation reactions that modulates gene activity [19]. Reduced folate is a natural and strong antioxidant crucial to maintaining intracellular oxidative stress and cell viability [20–22]. The vital roles of folate in modulating gene activity, intracellular oxidative stress, cell proliferation and tissue differentiation have led to the development of many drugs targeting folate-mediated one-carbon metabolism [23, 24]. The multi-activity of folate enzymes endows this vitamin as a modifiable environmental factor to prevent many important birth defects and associated diseases by dietary intervention.

Zebrafish (*Danio rerio*) is a vertebrate ideal for ontogenetic studies, including circulation [25, 26]. Possessing both *in vitro* convenience and *in vivo* complexity, zebrafish has emerged to be a powerful biological platform used in high-throughput studies for cardiovascular research. As in mammals, zebrafish possess a closed cardiovascular system with conserved cardiac components including; atria, ventricles, cardiac valves and a cardiac conduction system [27]. The genetic programs and molecular mechanisms involved in hematopoiesis, vasculogenesis and cardiac development in zebrafish are highly conserved [27–29]. In mammals, prenatal mortality resulting from experimentally induced heart defects could be uncontrollably high. Contrary to mammalian model animals, zebrafish embryos/larvae are able to survive till 5 to 7 days post fertilization (dpf) without a functional circulation system, allowing for the investigation and intervention that might lead to congenital circulation defects [30]. In addition, the embryos are optically transparent and develop quickly, allowing for noninvasive and continuous imaging throughout cardiac development. Zebrafish circulation system and related disease models have been extensively investigated. Supplementing with folic acid has also been shown to prevent the cardiac defects caused by toxic chemicals in zebrafish larvae, yet how folate affects development of zebrafish circulation system remains largely unexplored [31, 32].

We had developed a fluorescent transgenic line that displayed folate deficiency in a stage-, intensity-, and duration-controllable manner upon heat-shock induction [20]. A recombinant γ-glutamylhydrolase (γGH)-fused with a green fluorescent protein is induced by heat-shock induction, resulting in the conversion of folate polyglutamates to the monoglutamate form which are transported out of the cell. An impeded circulation system, along with other anomalies, was apparent in these FD larvae. In the current study, we characterized the circulation defects observed in these FD larvae and investigated the involved mechanisms. We determined the fluctuation of intracellular folate composition in developing FD embryos, which had not been fully addressed in previous studies. The impact of folate supplementation has also been examined and discussed.

## Materials and methods

### Materials

5-formyltetrahydrofolate (5-CHO-THF) was purchased from Schircks Laboratories (Bauma, Switzerland). Fetal bovine serum (FBS) and trypsin-EDTA were purchased from Invitrogen, Thermo Fisher Scientific Inc. (CA, USA). dNTP was purchased from FocusBio (CA, USA). The *in vitro* transcription kit, anti-DIG antibody, and NBT-BCIP used for WISH were purchased from Roche (Basel, Switzerland). The A375 human melanoma cells and A549 alveolar basal epithelial adenocarcinomic cells, originally from the American Type Culture Collection (ATCC), were purchased from the Bioresource Collection and Research Center (BCRC). Phospho-Histone H3 (pH3) antibody was from Santa Cruz Biotechnology Inc. (SC-374669, DA, USA). Goat anti-mouse IgG Alexa Flour^®^ 488 was from Abcam plc. (ab150113, Cambridge, UK). All other chemicals, including N-acetyl-L-cysteine (NAC), U0126, folic acid (FA), N-phenylthiourea and o-dianisidine were obtained from Sigma-Aldrich Chemical Co. (WI, USA). The HPLC gel filtration column AQUASIL C18 was from Thermo Fisher Scientific Inc. (MA, USA).

### Fish line and maintenance

The AB strain zebrafish and the transgenic line Tg(*gata1*:dsRed) expressing red fluorescent red blood cells were purchased from Taiwan Zebrafish Core Facility [33]. The zebrafish transgenic line Tg(*hsp*:EGFP-γGH), displaying inducible folate deficiency, was developed in our lab as described previously [20]. Zebrafish were maintained following the standard husbandry procedures [34]. Both the animal studies and the procedures performed were approved by Affidavit of Approval of Animal Use Protocol of National Cheng-Kung University (IACUC Approval NO. 103218).

### Induction of folate deficiency

Transgenic embryos with folate deficiency were generated and categorized as previously described [20]. In brief, Tg(*hsp*:EGFP-γGH) embryos were heat-shocked at 9 and 24 hours post fertilization (hpf) at 38–39°C for 1 hour each time and categorized into a control group (CTL, with no fluorescence), mild folate deficient group (MFD) and severe folate deficient group (SFD) based on their fluorescence intensity (ie. severity of folate deficiency) at 32 hpf. Previously, we had shown that the severity of folate deficiency was positively correlated to the intensities of heat-shock and green fluorescence.

### Compound treatment

Compounds, including 5-formyltetrahydrofolate (1 mM), folic acid (1 mM), U0126 (5 μM), N-acetyl-L-cysteine (20 μM) and dNTP (50 μM), were freshly prepared in embryo water, added directly to embryo water containing 0.003% N-phenylthiourea 30 minutes after heat-shock to reach the indicated concentrations and refreshed every day. Embryos were incubated in embryo water containing tested compounds at 28°C until observation.

### Hemoglobin staining

Hemoglobin was stained with o-dianisidine solution as previously described [35]. In brief, larvae at 3 dpf were anesthetized with 0.016% tricaine, followed by a 15-minute incubation in 0.6 mg/ml o-dianisidine containing 0.01 M sodium acetate (pH 4.5), 0.65% hydrogen peroxide, and 40% ethanol. Larvae were washed twice with 1× phosphate-buffered saline (PBS), fixed in 4% paraformaldehyde (PFA) and observed under a light microscope.

### Erythrocyte analysis

Erythrocytes of embryos generated by crossing Tg(*hsp*:EGFP-γGH) with Tg (*gata1*:dsRed) were analyzed by flow cytometry (BD Biosciences, FACSClibur). Dechorionated 2 dpf embryos were incubated in 1× trypsin-EDTA for 10 minutes at room temperature, followed by adding heat-inactivated FBS to a final concentration of 5% to stop the reaction. Cell suspensions were filtered through a 35-μm cell strainer and centrifuged at 200g for 4 minutes. The cell pellets were re-suspended in phosphate-buffered saline to a density of 5 × 10^6^ cells/ml before subjecting to flow cytometry for cell sorting and gating.

### Whole-mount in-situ hybridization and histochemical staining

Whole-mount *in situ* hybridization (WISH) with digoxigenin (DIG)-labeled riboprobes was performed following the protocol described by Jowett (2001) and Thisse (1993) [36, 37]. Riboprobes were generated by *in vitro* transcription in the presence of digoxigenin-11-UTP from linearized plasmid templates. Cryosectioning and H&E staining were performed following the protocols in the Zebrafish Book and as previously described [34, 38].

### Whole-mount immunostaining

Whole-mount immunostaining with phospho-Histone H3 (pH3) antibody (1:200) for proliferating cells was performed following the protocol described by Jowett (2001) and Thisse (1993) [36, 37]. In brief, embryos were fixed at indicated stages by 4% PFA/PBS and permeablized with acetone in -20°C. Embryos were incubated with mouse monoclonal pH3 antibody and goat anti-mouse IgG Alexa Flour^®^ 488 (1:400) sequentially with proper wash and observed under a fluorescent dissecting microscope.

### Cardiac function analysis

Larvae at 3 dpf were mounted in 3% methyl cellulose and video-recorded for cardiac pump (60 frames/sec) under a transmitted-light stereomicroscope (Leica, MDG28) equipped with a digital single-lens reflex camera (Canon, EOS 550D). End-systolic area and end-diastolic area were measured from the bright field time lapse image sequence analysis with a serial video stills with the online software ImageJ, an open platform for scientific image analysis (https://imagej.net/ImageJ). The cardiac stroke area, ejection fraction and cardiac output of larvae were calculated with the following equations: Stroke area = end diastolic area − end systolic area; Ejection fraction = stroke area / end diastolic area; Cardiac output = stroke area × heart rate.

### Analysis of folate derivatives

The intracellular content of embryonic folate adducts were measured as previously described [39]. In brief, approximately fifty embryos were homogenized in extraction buffer and then briefly heated in boiling water before centrifugation. Purified recombinant zebrafish γGH was added to the supernatant of extracts to hydrolyze folylpolyglutamates. After centrifugation and filtration, the clear supernatant was subjected to HPLC analysis.

### Homocysteine quantification

The homocysteine level of embryos was quantified using Axis^®^ Homocysteine Enzyme Immunoassay (AXIS-SHIELD, FHCY100) following the procedures described in the manufacture’s instruction. In brief, five to eight dechorionated embryos were homogenized in the sample pre-treatment solution to convert the reduced homocysteine to S-adenosyl-L-homocysteine (SAH). The SAH in sample were quantified by mouse anti-SAH antibody and the horse radish peroxidase (HRP)-conjugated anti-mouse antibody with the absorbance at 450 nm.

### Single embryonic cell migration

Plasmids (approximately 150–400 pg/embryos) encoding either EGFP or EGFP-γGH were injected into one single cell of the embryos at 64-cell stage and continuously recorded from 6 hpf to 7hpf with the frequency of 30 seconds per frame under a fluorescent dissecting microscope. The tested compounds, including 5-CHO-THF (1 mM) and NAC (20 μM), were added immediately to embryo water after injection. The serial images were processed and analyzed using Celltracker (v1.0) on MATLAB R2015a system [40]. Cells displaying comparable fluorescent intensity were selected and analyzed for their moving parameters.

### Reactive oxygen species assay

Accumulation of reactive oxygen species (ROS) in embryos was assessed using 2’,7’-dihydrodichlorofluorescein diacetate (H2DCFDA) staining as previously described [41]. The green fluorescent ROS signal was observed under a fluorescence dissecting microscope (excitation at 485 nm, emission at 560 nm).

### Wound healing assay

A375 human melanoma cells and A549 alveolar basal epithelial adenocarcinomic cells were cultured in Dulbecco’s Modified Eagle Medium containing 10% FBS at 37°C with 5% CO_2_. Cells were transfected with 4 μg of plasmid DNA expressing either EGFP or EGFP-γGH by electroporation. The transfected cells were seeded in culture plates coated with poly-D-lysine. A scratch was made with a pipet tip after cells attached properly. The wound area was recorded right after the scratch was made (OA) and 24 hours later (LA). The wound healing ability was quantified by the healed area after 24-hours of healing with the following equation: Healed area = OA − LA.

### Statistical analysis

The probability value (P value) was calculated with Student’s t test for cardiac function analysis and Mann-Whitney nonparametric U test for the rest at 95% confidence intervals using software GraphPad Prism 5.

## Results

### Impeded embryonic development and circulation system

**Fig 2:**
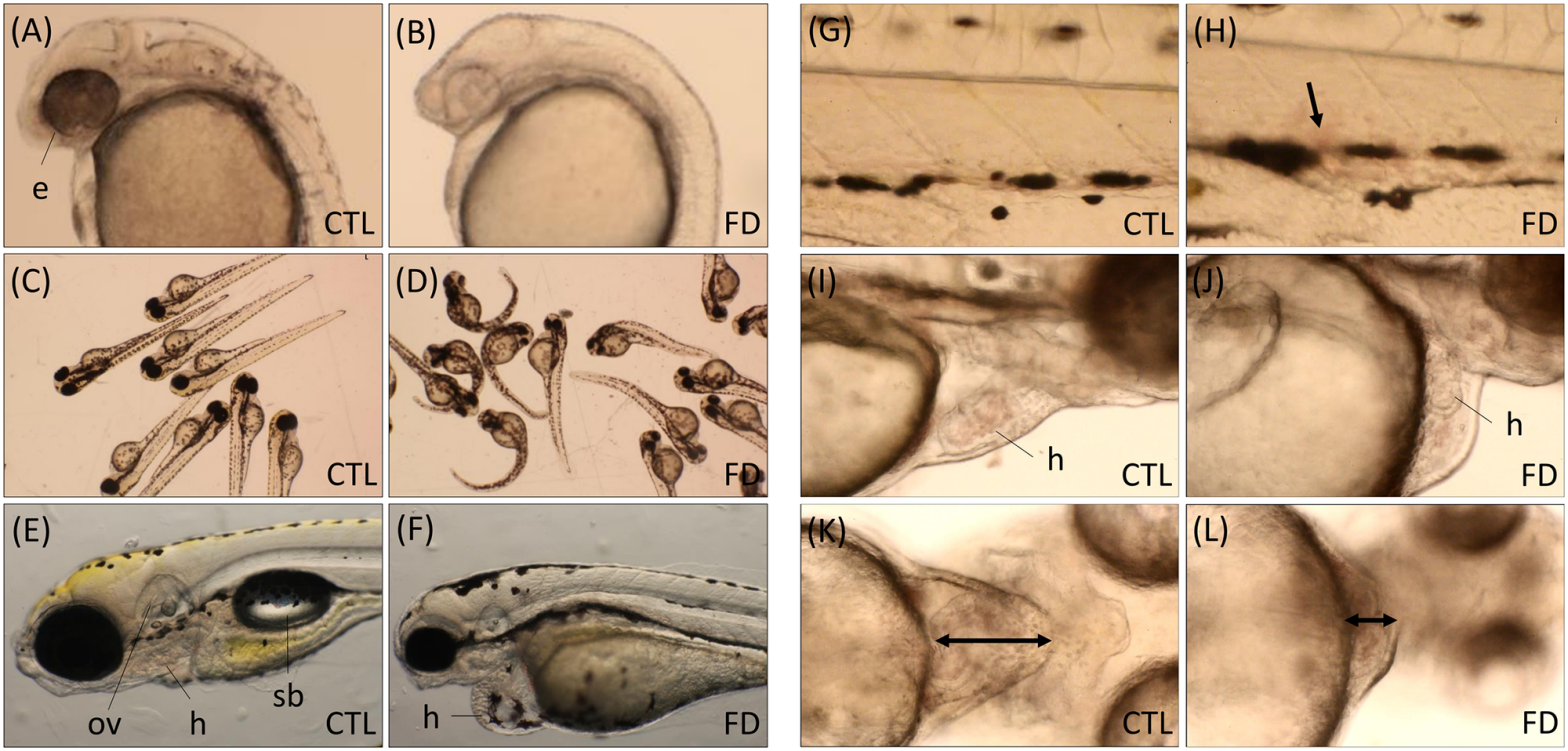
FD larvae displayed aberrant morphogenesis. FD larvae displayed morphological abnormalities in several tissues, including reduced pigmentation in both trunk and eyes (A, B), body curvature (C, D), shrunken eyes and otic vesicle, aberrant pericardial chamber and loss of swimbladder (E, F). Photos (A-F) and images (G-L; video stills of S1–S6 Videos) were taken when larvae reached 1 dpf (A, B), 3 dpf (C, D, I-L), 5 dpf (E, F) and 7 dpf (G, H). Ectopic accumulation of blood cells (arrow) (G, H) and pericardial chamber with altered dimensions (double-headed arrows) (K, L) were observed in FD larvae. CTL, heat-shocked non-fluorescent transgenic control; FD, folate deficiency; h, heart; e, eyes; sb, swim bladder; ov, otic vesicle.

Abnormalities, including smaller eyes, loss of pigmentation, body curvature, defective neural tissues, misshaped pericardial chamber and loss of swim bladder were seen in FD larvae (Fig 2A–2F). Significant slowing-down of blood flow and extravascular accumulation of red blood cells also appeared in FD larvae (Fig 2G and 2H; S1 and S2 Videos). Cardiac anomalies with diversified phenotypes, including pericardial edema, unsuccessful looping and distorted cardiac chambers with varied size were observed (Fig 2I–2L; S3–S6 Videos). The gross morphology of FD larvae indicate that folate deficiency impeded the development of the circulation system in FD larvae.

### Impaired hematopoiesis

**Fig 3:**
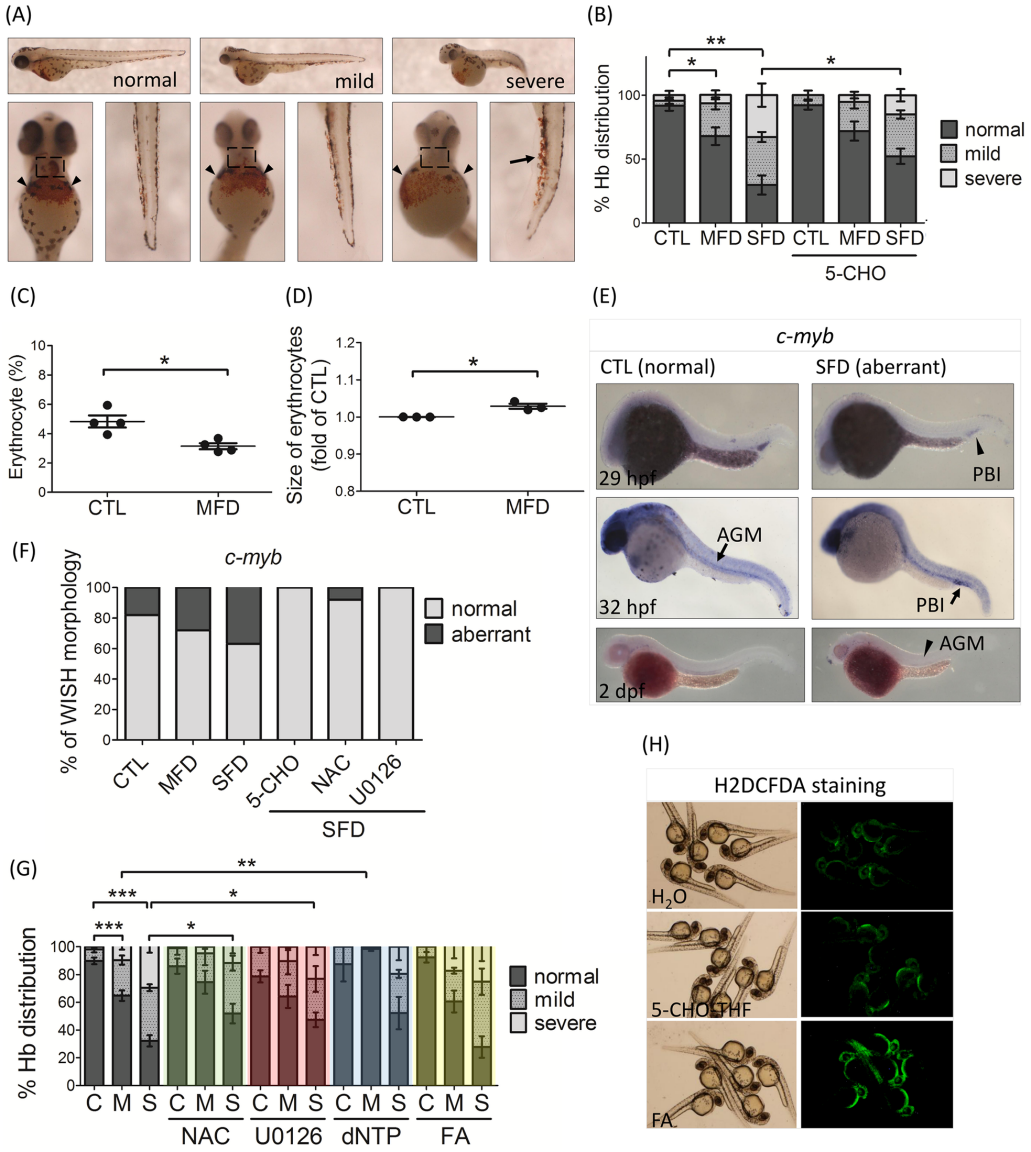
Zebrafish larval hematopoiesis and response to rescuing agents. (A, B) Hemoglobin of larvae in control and FD groups, with/without folate supplementation, were stained with o-dianisidine at 3 dpf. Hemoglobin signals were distributed most abundantly in the heart (dashed rectangles) and common cardinal veins (arrowheads) of control larvae (normal). Ectopic accumulation of hemoglobin in caudal veins (arrows) was often observed in FD larvae (mild and severe). The severity of anomalies was categorized and quantified based on the level and distribution of hemoglobin signals in larval heart and common cardinal veins. The images shown were the lateral (the upper panel) and ventral (the lower panel) views of larvae. Average of at least six independent experiments with the total sample number of 51–139 for each group are reported. (C, D) The relative number and size of embryonic erythrocytes were analyzed with flow cytometry for both control and FD embryos of 2-dpf generated by crossing Tg (hsp:EGFP-γGH) and Tg (gata1:dsRed). The numbers of erythrocytes were presented as the percentage of red fluorescent cells to total cell number. The size of erythrocytes was normalized with those of control larvae. Presented are data collected from at least three independent experiments with a total embryo number of approximately 30–40 for each group. (E) Hematopoiesis in both control and FD embryos was characterized by whole mount *in situ* hybridization with a riboprobe specific to *c-myb*, a hematopoietic stem cells marker. Reduced signals (arrowheads) with spatially and temporally altered distribution (arrows) were observed in embryos with severe folate deficiency. The larval responses to rescuing agents were quantified based on the distribution patterns of the *c-myb* signal at 32 hpf larvae (F) as shown in (E), and on the hemoglobin level (G) as shown in (A). There were approximately 10 to 40 larvae included for each group. (H) The 1-dpf wild-type larvae exposed to folic acid or 5-CHO-THF for 1 hour were examined for oxidative stress with H2DCFDA staining. C or CTL, heat-shocked non-fluorescent transgenic control; M or MFD, mild folate deficiency; S or SFD, severe folate deficiency; 5-CHO, 5-formyltetrahydrofolate; NAC, N-acetyl-L-cysteine; FA, folic acid. *, p<0.05; **, p<0.01; ***, p<0.001.

Decreased hemoglobin signals and red blood cells were observed in FD larvae, indicating interfered hematopoiesis. The hemoglobin stained with o-dianisidine was apparent and mainly distributed in the heart and common cardinal veins of control embryos, but was significantly and dose-dependently reduced in FD larvae (Fig 3A and 3B). Ectopic accumulation of hemoglobin was also observed occasionally around the caudal vein of larvae with severe folate deficiency. These abnormalities were prevented by supplementing with 5-formyltetrahydrofolate (5-CHO-THF), a reduced folate commonly used in anti-folate combinatorial therapy for leukemia, supporting the causal role of folate deficiency in observed hematopoietic defects (Fig 3B). To examine the number and size of erythrocytes, Tg(*hsp*:EGFP-γGH) was crossed with Tg(*gata1*:dsRed) to generate the double-transgenic larvae, which simultaneously displayed red fluorescent erythrocytes and folate deficiency upon heat-shock induction. The results of flow cytometric analysis on the dispersed red fluorescent erythrocyte suspension, prepared from the heterozygous larvae with mild folate deficiency at 2 dpf, revealed an approximately 35% decrease in the number and a significant increase in the size of erythrocytes, in comparison to control larvae (Fig 3C and 3D). The results of WISH showed that the distribution of *c-myb* transcripts, an essential transcription factor for hematopoiesis and a commonly used marker of hematopoietic stem cells, was altered spatially and temporally in FD embryos (Fig 3E and 3F) [42]. Hematopoietic development in vertebrates is constituted by two consecutive waves: the primitive wave followed by the definitive wave. In zebrafish, the tissues responsible for hematopoiesis shift from lateral mesoderm to caudal hematopoietic tissue and kidney marrow as the hematopoietic waves proceed [28]. The *c-myb* signal in the posterior blood island (PBI) in control embryos was enriched at 29 hpf and gradually diminished at 32 hpf. Then the signal became apparent in aorta-gonad-mesonephros (AGM) and remained obvious at 2dpf (Fig 3E). In FD embryos, the c-*myb* signal at PBI was weaker at 29 hpf, in comparison to control embryos, and enhanced at 32 hpf. At 2 dpf, the overall signal was faint at AGM of FD larvae (Fig 3E). These results indicate decreased hematopoietic stem cells and delayed hematopoiesis in FD embryos.

To examine the mechanism possibly involved in the FD-induced hematopoietic defects, several compounds were tested for their rescuing effectiveness. Both antioxidant N-acetyl-L-cysteine (NAC) and Erk inhibitor U0126 effectively rescued erythrocyte development (Fig 3F and 3G). Supplementing with dNTP also significantly prevented the hematopoietic anomalies. Unexpectedly, adding folic acid, the oxidized form of folate, failed to protect the embryos from FD-induced defective blood cell formation (Fig 3G). H2DCFDA vital staining revealed increased oxidative stress in wild-type embryos exposed to 1 mM folic acid for an hour, in comparison to untreated controls and those exposed to 5-CHO-THF (Fig 3H). These results indicate that increased oxidative stress, activated Erk signaling and nucleotide depletion contributed to the FD-induced impeded hematopoiesis.

### Impeded cardiogenesis

**Fig 4:**
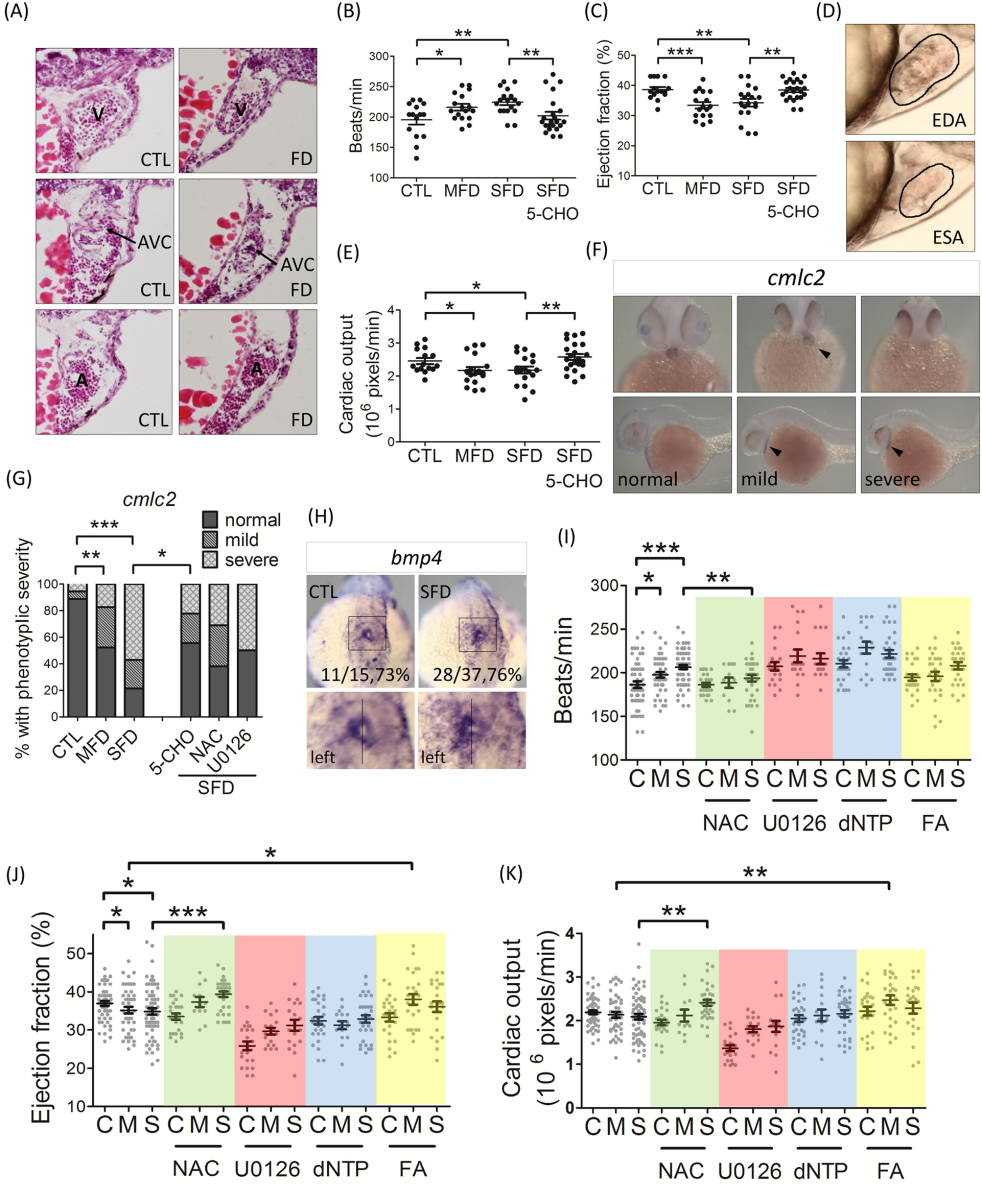
Zebrafish larval cardiogenesis and response to rescuing agents. (A) The serial cryo-sections of whole larvae at 3 dpf were HE-stained and examined for the integrity of heart chambers. (B-E) Larval heart beats were measured at 3 dpf. The cardiac ejection fraction and cardiac output were calculated from the dimension of cardiac chamber (D; circled area) in the video still images (lateral view with anterior to the right) as described in Materials and Methods. Data were collected from at least 3 independent experiments with the total sample number of 14–23 for each group. (F) The cardiac development in both control and FD larvae was characterized by WISH with the riboprobe specific to *cmlc2*, a cardiac primordium marker. Representative WISH images of *cmlc2* showed decreased or spatially altered (arrowheads) signal in FD larvae at 2 dpf. Images were taken from ventral view (upper panel) and lateral view with the head to the left (lower panel). (G) The severity of cardiac developmental anomalies observed in embryos with/without rescuing agents supplementation were categorized into normal, mild and severe and quantified based on the distribution pattern and intensity of the *cmlc2* WISH signal, as shown in (F). (H) The distribution of *bmp4* transcripts in embryos at 21 hpf was characterized by WISH. *Bmp4* expressed predominantly on the left side of embryonic cardiac disc in both control and FD embryos. Embryos were shown in dorsal view with head to the top and the boxed region magnified in inset at the lower panel. (I-K) The larval responses to rescuing agents were also evaluated based on cardiac function, including heart beats, cardiac ejection fraction and cardiac output on 3-dpf larvae exposed to the indicated compounds. Data were collected from at least 3 independent experiments with a total embryo number of 15–50 for each group. C or CTL, heat-shocked non-fluorescent transgenic control; FD, folate deficiency; M or MFD, mild folate deficiency; S or SFD, strong folate deficiency; A, atrium; AVC, atrium-ventricle canal; V, ventricle; EDA, end-diastolic area; ESA, end-systolic area; 5-CHO, 5-formyltetrahydrofolate; NAC, N-acetyl-L-cysteine; FA, folic acid. *, p<0.05; **, p<0.01; ***, p<0.001.

Serial cryostat sections prepared from FD larvae at 3 dpf revealed cardiac anomalies, including smaller chamber size, incorrect heart looping and a narrowed atrioventricular canal (Fig 4A). An approximately 12% increase was observed for the average heart rate in FD larvae, as compared to control larvae (Fig 4B). The alteration in the fraction of larval cardiac ejection and output were estimated from the difference between diastolic and systolic cardiac areas via the image analysis on serial video stills, as described in Materials and Methods (Fig 4D). FD larvae displayed a 12% decrease in ejection fraction and 11% decrease in cardiac output (Fig 4C and 4E). WISH results with the cardiac specific riboprobe *cmlc2* revealed incorrectly positioned heart primordium in FD larvae. In addition, the signal intensity was inversely and dose-dependently decreased, corresponding to the severity of folate deficiency (Fig 4F and 4G). Supplementing with 5-CHO-THF rescued all the above-mentioned cardiac abnormalities (Fig 4B, 4C, 4E and 4G), confirming the causal link between folate deficiency and impeded heart development in FD larvae.

The incorrect (opposite) positioning of the heart, observed in FD embryos, echoed structural and functional defects. These observations also implied a disturbed cardiac jogging and possibly left-right patterning during embryogenesis. Normally the symmetric heart cone, formed in the midline of zebrafish embryos, will undergo leftward displacement accompanied by rightward looping (cardiac jogging) during cardiac development, forming the ventricle in the anterior right side and the atrium in posterior left side [27]. However, no significant difference on the distribution pattern of *bmp4* transcripts (Fig 4H) and the development of Kuppfer’s vesicle (data not shown) was found between control and FD larvae. *Bmp4* is the signaling molecule responsible for the left-right patterning of the heart [43]. Kuppfer’s vesicle is a crucial organ mediating the initiation of zebrafish embryonic left-right patterning and the correct arrangement of brain, heart and gut [44]. These results indicate that folate deficiency does not affect the left-right symmetry of embryonic development.

Exposing FD embryos to NAC effectively prevented cardiac anomalies, supporting the contribution of increased oxidative stress to FD-induced heart defects (Fig 4I–4K). Adding folic acid also significantly alleviated the aberrant heart function. Unexpectedly, supplementing with dNTP and Erk inhibitor U0126 failed to protect the embryos from the harmful impact caused by folate deficiency. These results indicate that increased oxidative stress contributed to the FD-induced cardiac anomalies. However, disturbed nucleotide synthesis and activated Erk signaling might not take part in FD-induced heart defects, as we would have expected.

### Disturbed cell proliferation and intracellular one-carbon pools

**Fig 5:**
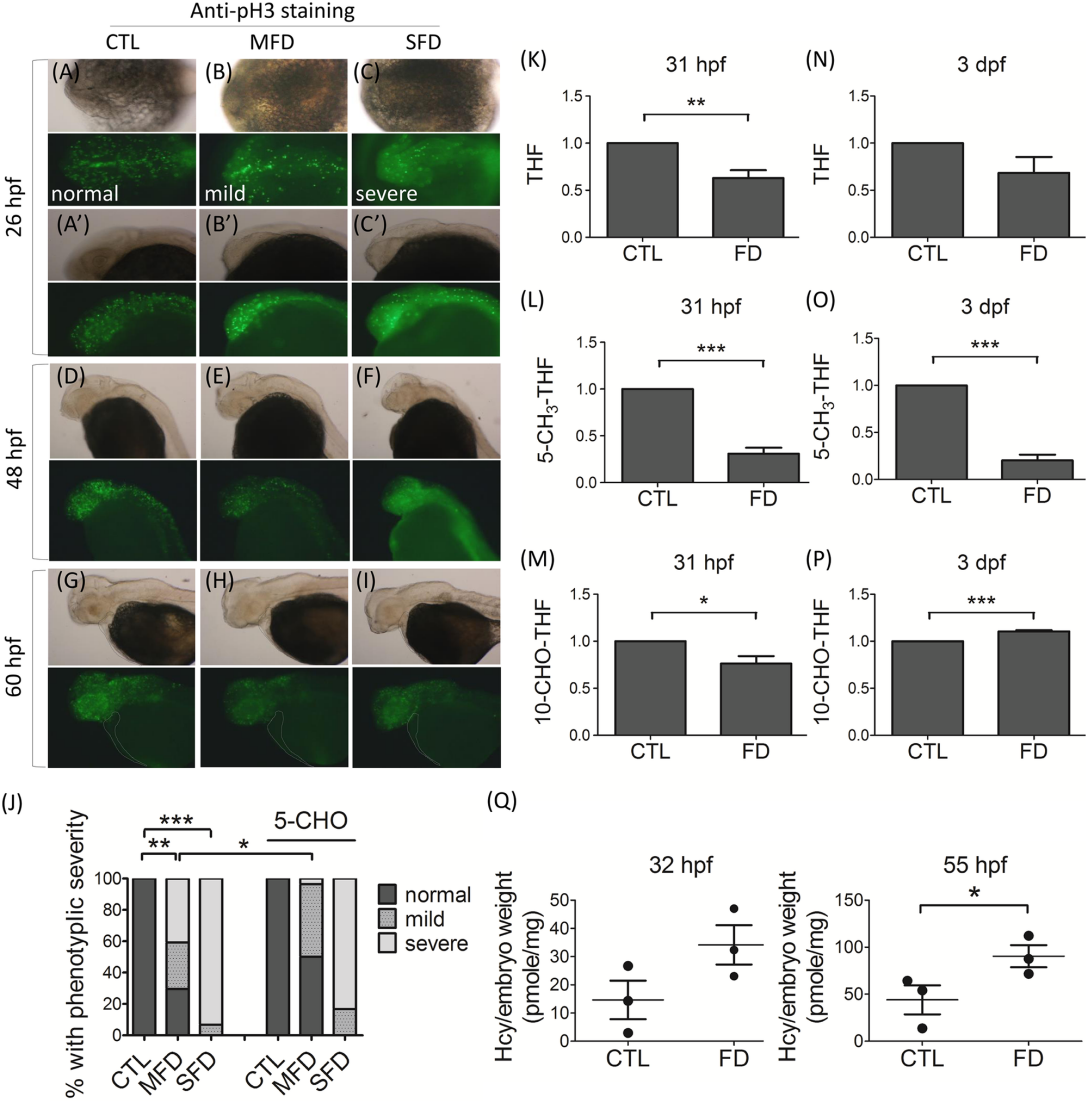
The impact of folate deficiency to embryonic cell proliferation and one-carbon pools. (A-I) Embryos at the indicated stages were subjected to whole mount immunological staining with anti-pH3 antibodies for proliferating cells in whole body and especially in the cardiac area (dashed line). The green fluorescent puncta represent the positive signal of pH3, which were distinctive and distinguishable from the homogeneous green fluorescent background raised from the heat-shock induced EGFP-γGH. Images were taken from dorsal view (A-C) and lateral view (A’-C’ and D-I) with anterior to the left. (J) Quantification of phenotypes (normal, mild, and severe) for embryonic cell proliferation in each group was based on the signal intensity detected at 26 hpf, as shown in (A-C). Data were collected from at least 3 independent experiments with the total sample number of 9–15 for each group. Larvae of 31 hpf (K-M) and 3 dpf (N-P) were subjected to intracellular folate content measurement with HPLC. (Q) Larvae of 32 hpf (left) and 55 hpf (right) were analyzed for homocysteine content. Data presented were the average of at least three independent trials and were normalized by embryo weights. CTL, heat-shocked wild-type control; MFD, mild folate deficiency; SFD, severe folate deficiency; FD, folate deficiency. 5-CHO, 5-formyltetrahydrofolate; THF, tetrahydrofolate; 10-CHO-THF, 10-formyltetrahydrofolate; 5-CH3-THF, 5-methyltetrahydrofolate. *, p<0.05; **, p<0.01; ***, p<0.001.

Decreased cell proliferation was observed at early stages for FD embryos. Considering the essentialness of folates in nucleotide biosynthesis, it is conceivable to hypothesize that folate deficiency disturbed intracellular dNTP pools, hampering cell proliferation. Results from whole-mount immunostaining with anti-pH3 antibodies, a mitosis marker, revealed significantly decreased cell proliferating signals in FD embryos at 26 hpf. The fluorescent dots, representing positive signals, were apparent in the head region and anterior trunk of control larvae, but were decreased in number in FD embryos (Fig 5A–5C and 5A’–5C’). The decreased signal of cell proliferation in FD embryos was ameliorated by supplementing with 5-CHO-THF, confirming the causal role of folate deficiency (Fig 5J). The signals observed in FD larvae at 48- and 60-hpf were also weaker than those in control larvae of the same stages (Fig 5D–5I). Nevertheless, we noticed that the difference in the signal intensity between control and FD larvae of the same stages gradually diminished as embryonic development proceeded. We also noticed that no apparent anti-pH3 signal was observed in the heart region of larvae at 60 hpf for all control and FD larvae (Fig 5G–5I). No appreciable signal for apoptotic cells was revealed in both control and FD larvae at 26, 48 and 60 hpf examined with the TUNEL assay either (data not shown). Together, with the different rescuing effects provided by dNTP to blood cells and heart development, these results show that FD disturbed nucleotides synthesis and hampered embryonic cell proliferation, which exerted a more profound impact to hematopoiesis compared to cardiogenesis.

Incoherent fluctuation among folate adducts was observed in FD embryos as embryonic development proceeded. The intracellular folate derivatives of both control and FD embryos at early stages were measured with HPLC. Our data revealed that both tetrahydrofolate (THF) and 5-methyltetrahydrofolate (5-CH_3_-THF) were decreased in FD larvae of 31-hpf and 3-dpf, with a more significant decrease observed for 5-CH_3_-THF (Fig 5K, 5L, 5N and 5O). Interestingly, 10-formyltetrahydrofolate (10-CHO-THF) was decreased at 31 hpf, but increased at 3 dpf in FD larvae, in comparison to the control at the same stages (Fig 5M and 5P). THF is the basic moiety as the one-carbon carrier. 5-CH_3_-THF and 10-CHO-THF are the two folate adducts crucial for epigenetic control and cell proliferation, since they directly participate in the formation of SAM and nucleotides, respectively. The level of homocysteine was also increased in FD larvae, echoing the significant decrease of intracellular 5-CH_3_-THF (Fig 5Q). Elevated homocysteine levels is an indicator of folate deficiency, especially 5-CH_3_-THF, and an independent risk factor for cardiovascular abnormality. Our results indicate that different folate derivatives fluctuated differentially in response to folate deficiency.

### Impeded cells migration

**Fig 6:**
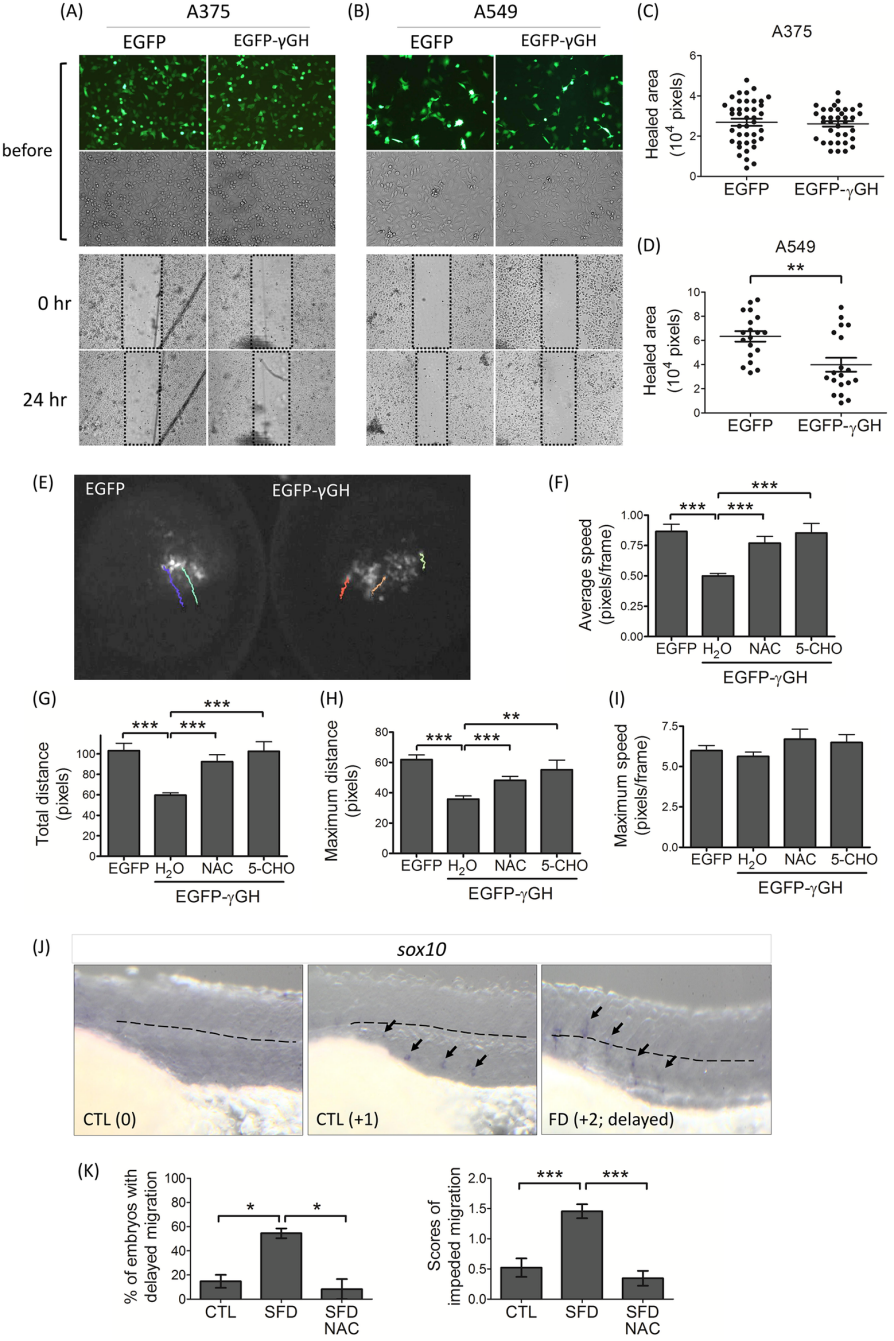
Folate deficiency impeded embryonic cells migration. The cultured A375 human melanoma cells (A) and A549 alveolar basal epithelial adenocarcinomic cells (B) were transfected with plasmids expressing either EGFP or EGFP-γGH fusion protein and subjected to the wound-healing assay. The wound was scratched and recorded for the scratched area immediately (0 hr) and again one day later (24 hr). The migration of A375 (C) and A549 (D) cells was evaluated by the “healed area”, which was calculated as described in Materials and Methods. Presented are the data collected from approximately 20 different wound areas from at least 3 to 6 independent repeats. (E) Wild-type zebrafish embryos at 64-cell stage were injected with the plasmids expressing either EGFP or EGFP-γGH into one single cell and continuously recorded for the migration of injected cells following the green fluorescence. Each colored line represents a single cell migratory track in one-hour recording period. The migratory parameters of recorded cells, including average speed (F), total distance (G), maximum distance (H) and maximum speed (I), were calculated with the on-line software CellTracker (v1.0, F. Piccinini et al., 2015) on MATLAB R2015a system. Zebrafish larvae of 31 hpf were subjected to WISH with the riboprobe specific to *sox10* to track the migration of neural crest cells. (J, K) The extent of neural crest cells (arrows) migration in FD embryos with/without NAC exposure was graded and quantified based on the following criteria: completely evacuated from the neural crest (0), still visible and below (+1) or above (+2, delayed) the trunk mid-line (dotted line). CTL, heat-shocked non-fluorescent transgenic control; FD, folate deficiency; NAC, N-acetyl-L-cysteine; 5-CHO, 5-formyltetrahydrfolate. *, p<0.05; **, p<0.01; ***, p<0.001.

Diversity in the impact to cell migration due to folate deficiency was revealed in both *in vivo* and *in vitro* studies. Cells transfected with plasmids carrying EGFP, or EGFP-γGH fusion cDNAs to induce folate deficiency, were subjected to wound healing assays (Fig 6A and 6B). The results showed that the migration of A375 human melanoma cells were not affected by overexpressed EGFP-γGH; whereas significantly decreased migratory activity was observed in FD A549 alveolar basal epithelial adenocarcinomic cells (Fig 6C and 6D). The same plasmids, expressing EGFP or EGFP-γGH fusion proteins, were also injected into a single cell of wild-type embryos at 64-cell stage to simultaneously induce folate deficiency and labeled the individual affected blastomeres with green fluorescence. The time-lapse video recording for cell migration showed that the control cells overexpressing EGFP moved downward unanimously and steadily to the vegetal pole (Fig 6E; S7 Video, left). However, the blastomeres overexpressing EGFP-γGH displayed decreased moving activity and the loss of directional migration in which the migratory path and direction were scattered (Fig 6E; S7 Video, right). The average speed, total traveling distance and maximum moving distance were all significantly decreased in the cells expressing EGFP-γGH (Fig 6F–6H). No significant difference was observed for the maximum speed of movement (Fig 6I). All the above anomalies were prevented by supplementing with either 5-CHO-THF or NAC.

Impeded migration of neural crest cells, the embryonic heart progenitors, was observed in developing FD embryos. Normally, most of the neural crest cells had evacuated from the dorsal margin of the closing neural tube and migrated to distant sites by 2 dpf. However, the migratory neural crest cells in FD embryos were still present at the bilateral bands around the embryonic mid-line (Fig 6J). This significant delay of neural crest cell migration observed in FD embryos was also rescued by supplementing with NAC (Fig 6K), supporting the causal role of increased oxidative stress in the FD-induced impeded neural crest cells migration.

### Intact vascular development

The extravascular accumulation of red blood cells, observed in FD larvae, implied altered vascular integrity and possibly malformed vessels. The development of vasculature was examined with WISH using the riboprobes specific to vessels (*fli1a*), veins (*flt4*) and arteries (*notch3* and *vegfaa*). The results showed no appreciable difference between control and FD embryos for all four probes employed (S1 Fig). These results suggested that folate deficiency did not affect vascular development. In addition, the slowed-down blood flow and ectopic accumulation of RBC observed in FD larvae were not due to impeded vessels development.

## Discussion

In the current report, we show that FD larvae display impeded hematopoiesis and cardiac development, the pathological characteristics often observed in patients with FD. We observed no apparent abnormality in vasculogenesis. Our results suggest that increased oxidative stress was involved in both FD-induced hematopoietic and cardiac development. These results add an additional cause for FD-induced developmental circulation defects. These results are also conceivable since reduced folate has been documented for their strong anti-oxidative activity in several recent studies. What was unexpected in this study, and less addressed in the literature, are the findings of: incompetence of folic acid supplementation to rescue hematopoietic anomalies; different sensitivity to folate deficiency displayed among tissues; incoherent fluctuation of embryonic folate status in response to folate deficiency; diverse mechanisms involved in FD-induced pathologies. Our studies support the importance of folate to embryonic circulation development and provide a possible explanation for the conflicting effects observed clinically in patients suffering from FD and/or receiving folate supplementation. Our results also support the properness of using this model for further investigation on FD-induced circulation defects.

Supplementing FD zebrafish with folic acid protected heart development but failed to prevent hematopoietic defects. This was unexpected. Folic acid is the oxidized form of folate commonly included in folate fortified food and vitamin supplements available to the general public for daily nutrient supplementation and to individuals with special needs, such as folate deficiency or pregnancy. Folic acid is the most stable form of folate, but the least potent antioxidant, in comparison to all naturally existing reduced folates [21]. Folic acid has even been demonstrated for its potential of acting as a ROS generator, although the effect of decreasing oxidative stress in experimental animal and cells has also been reported [45, 46]. We had previously shown that reduced folate alleviated ethanol-induced oxidative stress in zebrafish larvae, but in the current studies we observed increased oxidative stress in the embryos exposed to folic acid [41]. It appears that folic acid acts as a double-edged sword where it can serve as the one-carbon carrier in folate-mediated one-carbon metabolism, but also has the potential to increase oxidative stress, likely depending on whether the supplemented folic acid can be reduced properly before reaching its acting targets. This might be why the contradictory effects of folic acid supplementation in preventing diseases, including congenital heart disorders, were sometime observed clinically and in model animals [3–10].

Our data indicate that different folate derivatives fluctuate differentially in response to intracellular FD. This incoherent fluctuation among different folate derivatives reflects the distinct biochemical activity of each folate derivative and the complexity of folate-mediated one-carbon metabolism. It adds to the complexity of diverse tissue sensitivity to FD. One would expect that all folate derivatives would decrease concurrently upon folate deficiency. It was peculiar that the level of 10-CHO-THF in FD larvae was decreased initially, but restored and even increased later as embryogenesis continued; whereas 5-CH_3_-THF remained significantly and persistently lower, in comparison to control embryos at the same stages. Folate is crucial for cell proliferation since 10-CHO-THF and 5,10-CH_2_-THF are required for the biosynthesis of purine and dTMP, respectively. The successful rescuing with dNTP for defective blood cell formation and the decreased cell proliferation in FD embryos support the speculation that folate deficiency disturbs nucleotide formation, hampers cell proliferation, and hence hematopoiesis. This is also in agreement with our current knowledge that FD-induced macrocytic anemia was most likely caused by impaired DNA synthesis [47]. The initially decreased, and then progressively increased 10-CHO-THF in FD developing embryos, suggests the recovery of dNTP formation leading to the gradually vanished difference in cell proliferation between control and FD growing larvae. These results also reveal a stage-dependent variation of embryonic folate homeostasis during embryogenesis.

Intracellular 5-CH_3_-THF, on the other hand, was constantly and significantly lowered in developing FD embryos. That dNTP rescues hematopoietic anomalies but not cardiac defects, suggests that a mechanism other than impaired nucleotide synthesis and cell proliferation was involved in improper heart development. Heart development in zebrafish proceeds through steps similar to those of other vertebrates. It begins with the specification of pre-cardiac cells in very early embryogenesis in the anterior lateral plate mesoderm. Extensive migration and differentiation of cardiac neural crest cells then follow subsequently and contribute to the formation of the myocardium of the primitive heart tube, the endothelium of the ventral aorta and the bulbus arteriosus [30]. Neural crest ablation has been shown to result in numerous cardiac maldevelopment in zebrafish, including reduced heart rate, defective myocardial maturation and failed recruitment of progenitor cells from the second heart field, similar to those observed in FD-embryos [48]. Epigenetic modification is central to the control of cardiac progenitor cells commitment during heart development [49]. Intracellular methylation mediated by SAM constitutes a crucial element in epigenetic control network. Increased homocysteine and decreased 5-CH_3_-THF, the methyl group donor for SAM formation, implies a disturbed cellular methylation potential in FD embryos. Previous studies have shown that nucleotide biosynthesis was preserved in FD at the expense of homocysteine remethylation and SAM biosynthesis [50, 51]. Together, with the observation of impeded blastomeres and neural crest cells migration, our results suggest a possibility that aberrant embryonic cell migration, likely related to the disturbed intracellular methylation potential, took part in the FD-induced cardiogenic defects.

Increased oxidative stress contributes to FD-induced pathogenesis. Results that supplementing with antioxidant NAC significantly prevented the FD-induced cardiac and hematopoietic deficits unveiled an additional mechanism for the FD-induced heart and blood cells anomalies. Reduced folates are strong antioxidants and can act as free radical scavengers [21, 52]. In addition, the majority of intracellular NADPH is generated via folate-mediated one-carbon metabolism [22]. We have shown previously that folate is vital for maintaining embryonic oxidative stress and intracellular reducing power [41]. Hematopoietic stem cells and erythroid maturation are extremely sensitive to increased oxidative stress and intracellular redox status [53]. A positive correlation between elevated oxidative stress and the occurrence of congenital heart disease has also been reported [54, 55]. Our data support the previous findings and provide an additional rationale for the preventive effects of folate supplementation to FD-induced anemia and heart diseases and adds more emphasis to the importance of maintaining proper oxidative stress in developing embryos.

There are several questions unveiled in the current study that warrant further investigation. First, how the intracellular level of 10-CHO-THF is regulated, in response to folate deficiency, to preserve cell proliferation and enable embryogenesis is not clear. The incoherent fluctuation among folate derivatives during embryogenesis may provide flexibility and enable the cells/tissues to deal with various challenges during organogenesis (ex. High proliferating hematopoietic cells vs. active migrating neural crest cells) and to respond differentially to cell stress (ex. imbalanced one-carbon pool and increased oxidative stress). Our results echo the viewpoint that it is folate utilization, rather than environmental folate levels, that determines the preventive effects against diseases [4]. Also, how folate modulates embryonic cells migration *in vivo* remains elusive. We have shown previously that the expression of N-cadherin was decreased in FD-embryos, which might contribute to defective neural tissue development [20]. Nevertheless, both A375 and A549 cells express N-cadherin, but displayed cell-type dependent migration in our *in vitro* study [56, 57]. These observations, support the diverse sensitivity and response to folate status among different cell types, but also suggest that other molecule, besides N-cadherin, might be involved in modulating the migratory activity of cells in response to folate deficiency. The role and regulation of N-cadherin and/or any other molecule in the FD-induced impaired cell migration will be investigated.

**Fig 7:**
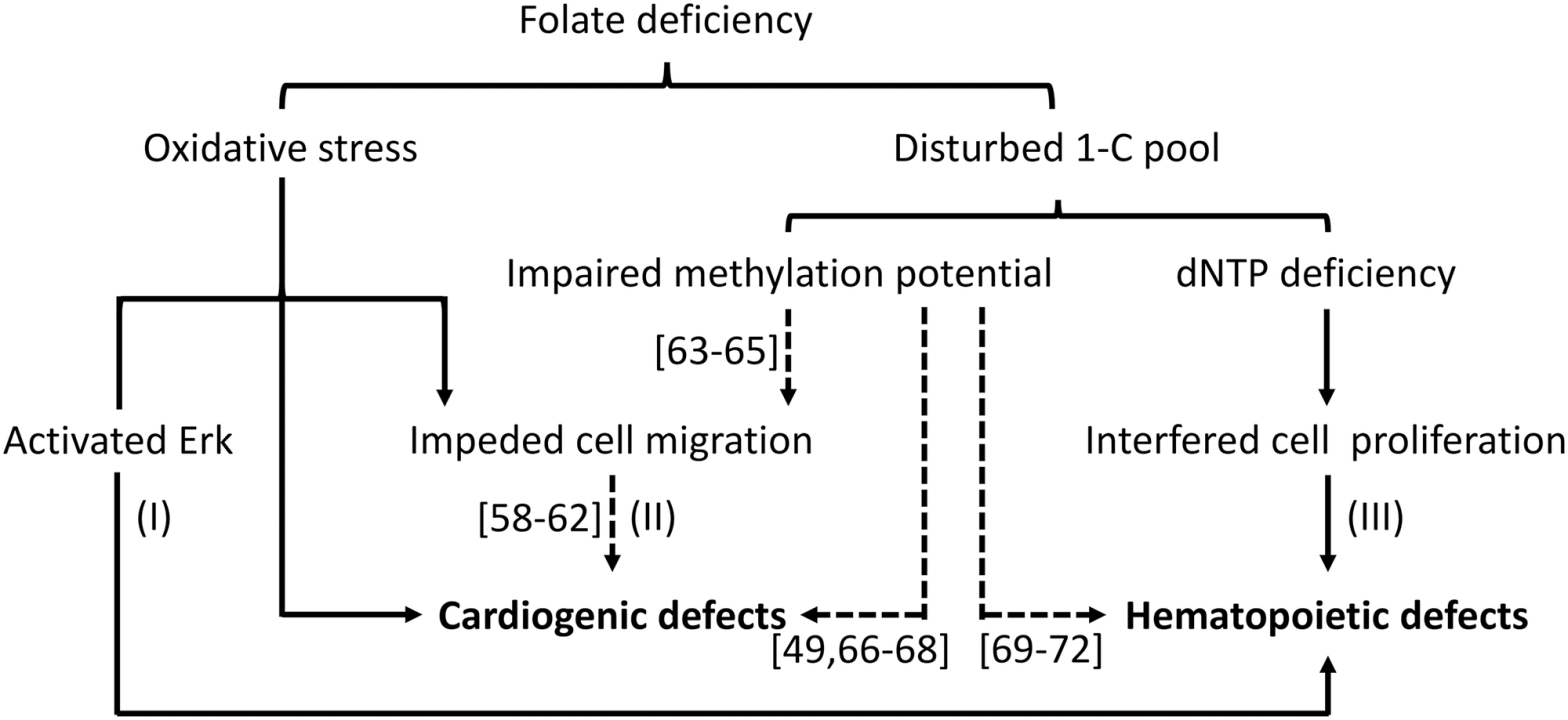
Prospective causal links involved in FD-induced developmental defects of the zebrafish circulation system. FD increases embryonic oxidative stress, leading to activated Erk (I) and impeded cell migration (II), which contributed to impeded hematopoiesis and cardiogenesis, respectively. FD also disturbed one-carbon metabolism, which interferes with nucleotide synthesis (III) and cell proliferation/hematopoiesis. The impeded one-carbon metabolism may also disturb intracellular methylation potential, which hampers primordial cell migration (II), leading to cardiogenic defects. This scheme is depicted based on the results reported in the current study (solid lines) and those in the literature (dashed line).

Based on the results of the current study, and those reported by other researchers, a potential pathomechanism underlying FD-induced circulation defects is depicted (Fig 7). FD (1) increased oxidative stress and caused both hematopoietic and cardiac defects via different molecular mechanisms, where activated Erk (I) and impeded cell migration (II) might be involved, respectively. FD also (2) disturbed nucleotides biosynthesis, hampered cell proliferation (III) and led to hematopoietic defects. Disturbed 1-C pool might also disturb intracellular methylation potential, causing profound impact to the activity of genes involved in regulating cell migration (II) and leading to cardiac defects [49, 58–72]. The tissue-specific response to altered folate status might reflect the physiological status and vulnerability of target cells and provide an explanation for the conflicting results observed clinically in patients suffering from FD and/or receiving folate supplementation. The potential of folic acid in modulating cellular oxidative stress should be further examined and taken into consideration when anti-folate agents and folate supplementation are administrated for diseases prevention or therapy. Our data also supports the use of this model for studying folate-related physiology, including the modulation of cell size and migration, cardiogenesis, hematopoiesis and the efficacy of folate supplementation.

## Supporting information

S1 Video The blood flow of a control larva. The blood flow in the mid-trunk region of a 7-dpf control larva mounted in 3% methyl cellulose was video-recorded in lateral view with anterior to the left.
(AVI)

S2 Video The blood flow of a folate deficient larva. The blood flow in the mid-trunk region of a 7-dpf folate deficient larva mounted in 3% methyl cellulose was video-recorded in lateral view with anterior to the left.
(AVI)

S3 Video The cardiac pumping of a control larva. The cardiac pumping of a 3-dpf control larva mounted in 3% methyl cellulose was video-recorded in lateral view with anterior to the right.
(MOV)

S4 Video The cardiac pumping of a folate deficient larva. The cardiac pumping of a 3-dpf folate deficient larva mounted in 3% methyl cellulose was video-recorded in lateral view with anterior to the right.
(MOV)

S5 Video The dorsal view of cardiac chamber of a control larva. A control larva at 3-dpf was mounted in 3% methyl cellulose and video-recorded from dorsal view with anterior to the right.
(MOV)

S6 Video The dorsal view of cardiac chamber of a folate deficient larva. A folate deficient larva at 3-dpf was mounted in 3% methyl cellulose and video-recorded from dorsal view with anterior to the right.
(MOV)

S7 Video Blastomeres migration in embryos. Plasmids encoding either EGFP (left) or EGFP-γGH (right) were injected into one single cell of wild-type embryos at 2 hpf. The migratory tracks of green fluorescent blastomeres were continuously monitored from 6 hpf to 7hpf.
(MOV)

S1 Fig Vascular development in FD larvae. Embryos of both control and folate deficiency at indicated stages were subjected to WISH with the riboprobes *fli1a* for vessels (A-C), *flt4* for vein (D-F), and *notch3* (G, H) and *vegfaa* for artery (I-K). Larvae were shown with anterior to the left in lateral view (A-K; upper panel) and dorsal view (A’-K’; lower panel). The numerator and denominator at the lower right-hand corner indicate the number of larvae exhibiting the displayed phenotype and the total larvae in the group, respectively. CTL, heat-shocked non-fluorescent transgenic control; MFD, mild folate deficiency; SFD, severe folate deficiency.
(TIF)

S1 File The primary data underlying results. The primary data underlying each result are provided and summarized in table format. The correspond figures of each table are footnoted.
(PDF)
