# Supplementary figures and images for: One crisis, diverse impacts—Tissue-specificity of folate deficiency-induced circulation defects in zebrafish larvae
Source: PLoS One. 2017 Nov 27;12(11):e0188585. doi: 10.1371/journal.pone.0188585 (PMC5703520; doi:10.1371/journal.pone.0188585)

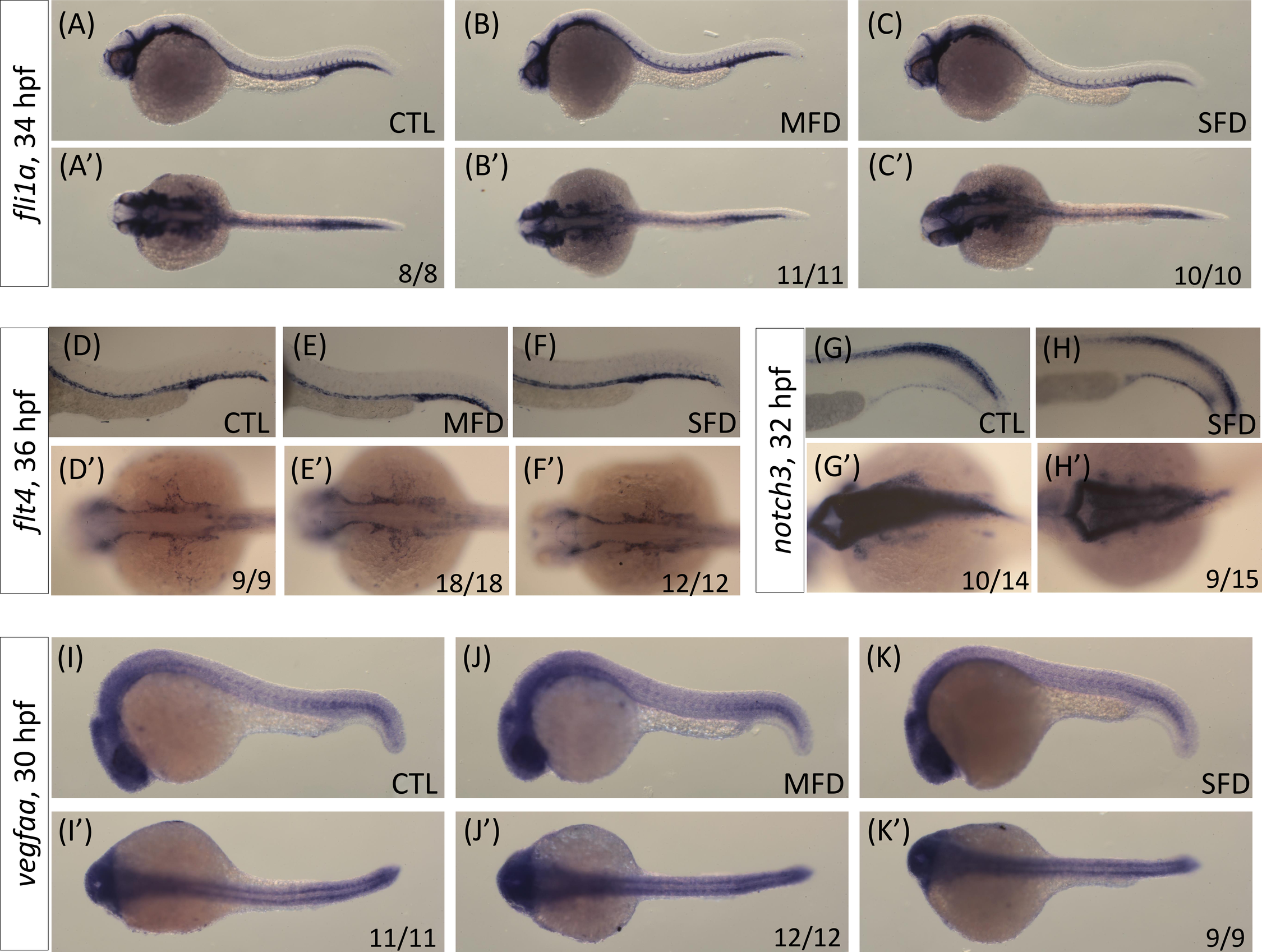

Supplement: S1 Fig — Embryos of both control and folate deficiency at indicated stages were subjected to WISH with the riboprobes fli1a for vessels (A-C), flt4 for vein (D-F), and notch3 (G, H) and vegfaa for artery (I-K). Larvae were shown with anterior to the left in lateral view (A-K; upper panel) and dorsal view (A’-K’; lower panel). The numerator and denominator at the lower right-hand corner indicate the number of larvae exhibiting the displayed phenotype and the total larvae in the group, respectively. CTL, heat-shocked non-fluorescent transgenic control; MFD, mild folate deficiency; SFD, severe folate deficiency. (TIF) [file pone.0188585.s008.tif]
