# Supplementary material for: One crisis, diverse impacts—Tissue-specificity of folate deficiency-induced circulation defects in zebrafish larvae
Source: PLoS One. 2017 Nov 27;12(11):e0188585. doi: 10.1371/journal.pone.0188585 (PMC5703520; doi:10.1371/journal.pone.0188585)
Supplement: S1 File — The primary data underlying each result are provided and summarized in table format. The correspond figures of each table are footnoted. (PDF) [file pone.0188585.s009.pdf]

Hb distribution pattern (mean; SD)

Treatment	E3 buffer (embryo water)			5-CHO-THF		
Larvae	CTL	MFD	SFD	CTL	MFD	SFD
Normal (%)	91.67; 9.65	67.83; 16.87	29.67; 18.48	92.17; 8.61	71.83; 18.4	52.17; 14.68
Mild (%)	4; 6.23	25.67; 11.38	37.5; 9.649	7.83; 8.61	22.83; 12.66	32.67; 8.12
Severe (%)	4.33; 8.04	6.67; 8.57	32.83; 22.28	0; 0	5.33; 6.377	15; 11.95

This table corresponds to Fig 3B

Erythrocyte number and size (mean; SD)

Larvae	CTL	MFD
Erythrocyte number to total cell number (%)	4.83; 0.83	3.15; 0.41
Size of erythrocyte (Fold of CTL)	1.00; 5.528e-007	1.03; 0.01

This table corresponds to Figs 3C and 3D

c-myb expression

Treatment	E3 buffer (embryo water)			5-CHO-THF	NAC	U0126
Larvae	CTL	MFD	SFD	SFD	SFD	SFD
Normal (%)	82	72	63	100	92	100
Aberrant (%)	18	28	38	0	8	0

This table corresponds to Fig 3F

Hb distribution pattern (mean; SD)

Treatment	E3 buffer (embryo water)			NAC		
Larvae	CTL	MFD	SFD	CTL	MFD	SFD
Normal (%)	89.86; 11.24	64.82; 17.7	32.23; 18.95	86; 12.19	74.5; 23.46	51.88; 20.02
Mild (%)	8.27; 10.1	25.55; 15.4	38.27; 11.53	13; 10.65	20.75; 23.94	36.38; 15.17
Severe (%)	1.86; 4.8	9.68; 10.15	29.68; 20.72	1; 2.24	4.75; 4.17	12; 14.27
Treatment	U0126			dNTP		
Larvae	CTL	MFD	SFD	CTL	MFD	SFD
Normal (%)	78.67; 7.51	64.2; 18.59	47.4; 11.87	87.5; 25	98.5; 3	52.25; 23.34
Mild (%)	21.33; 7.51	25.4; 20.91	29.4; 20.72	12.5; 25	1.5; 3	28.25; 5.74
Severe (%)	0; 0	10.4; 5.32	23.2; 13.35	0; 0	0; 0	19.5; 19.33
Treatment	Folic acid					
Larvae	CTL	MFD	SFD			
Normal (%)	92.4; 8.3	60.6; 17.64	27.8; 17.3			
Mild (%)	7.6; 8.3	22.2; 4.76	47; 21.14			
Severe (%)	0; 0	17.4; 19.05	25.4; 23.45			

This table corresponds to Fig 3G

Cardiac function analysis (mean; SD)

Treatment	E3 buffer (embryo water)			5-CHO-THF
Larvae	CTL	MFD	SFD	SFD
Heart rate (Beats/min)	195.20; 29.38	216.00; 22.15	224.30; 21.10	202.10; 29.94
Ejection fraction (%)	38.60; 3.25	33.41; 4.42	34.26; 5.46	38.43; 3.36
Cardiac output (10^6 pixels/min)	2.45; 0.36	2.17; 0.45	2.18; 0.45	2.58; 0.43

This table is corresponds Figs 4B, 4C, 4E

cmlc2 expression

Treatment	E3 buffer (embryo water)			5-CHO-THF	NAC	U0126
Larvae	CTL	MFD	SFD	SFD	SFD	SFD
Normal (%)	88.89	52.17	21.43	55.56	38	50
Mild (%)	5.56	30.43	21.43	22.22	31	0
Severe (%)	5.56	17.39	57.14	22.22	31	50

This table corresponds to Fig 4G

Cardiac function analysis (mean; SD)

Treatment	E3 buffer (embryo water)			NAC		
Larvae	CTL	MFD	SFD	CTL	MFD	SFD
Heart rate (Beats/min)	186.3; 27.24	197.6; 21.18	206.4; 19.71	186.2; 11.42	188.5; 20.70	193.6; 22.07
Ejection fraction (%)	36.93; 4.53	35.16; 5.98	34.82; 6.86	33.48; 4.05	37.33; 4.56	39.33; 4.21
Cardiac output (10^6 pixels/min)	2.19; 0.34	2.14; 0.45	2.09; 0.52	1.96; 0.31	2.12; 0.47	2.41; 0.4
Treatment	U0126			dNTP		
Larvae	CTL	MFD	SFD	CTL	MFD	SFD
Heart rate (Beats/min)	207.3; 21.89	219.3; 30.54	216.0; 25.63	210.2; 21.26	228.8; 27.35	221.6; 24.71
Ejection fraction (%)	25.86; 5.24	29.67; 3.77	31.22; 6.12	32.41; 5.17	31.25; 4.19	32.84; 5.54
Cardiac output (10^6 pixels/min)	1.37; 0.31	1.8; 0.28	1.87; 0.51	2.05; 0.42	2.12; 0.54	2.16; 0.46
Treatment	Folic acid					
Larvae	CTL	MFD	SFD			
Heart rate (Beats/min)	194.5; 18.04	195.8; 26.83	208.1; 21.26			
Ejection fraction (%)	33.29; 5.41	38; 7.01	36.09; 6.67			
Cardiac output (10^6 pixels/min)	2.22; 0.49	2.47; 0.54	2.29; 0.6			

This table corresponds to Figs 4I-4K

PH3 signal

Treatment	E3 buffer (embryo water)			5-CHO-THF		
Larvae	CTL	MFD	SFD	CTL	MFD	SFD
Normal (%)	100	29.6	0	100	50	0
Mild (%)	0	29.6	6.7	0	46.4	16.7
Severe (%)	0	40.7	93.3	0	3.6	83.3

This table corresponds to Fig 5J

Intracellular folate derivatives (mean; SD)

Folate derivatives	THF		$5\text{-}CH_3\text{-}THF$		10-CHO-THF	
larvae	CTL	FD	CTL	FD	CTL	FD
31 hpf (Fold of CTL)	1; 3.918e-007	0.63; 0.2	1; 4.768e-007	0.31; 0.13	1; 4.298e-007	0.76; 0.18
3 dpf (Fold of CTL)	1; 4.768e-007	0.68; 0.34	1; 4.768e-007	0.20; 0.12	1; 5.528e-007	1.11; 0.02

This table is corresponds to Figs 5K-5P

Homocysteine level (Homocysteine/embryo weight) (mean; SD)

larvae	CTL	FD
32 hpf (pmole/mg)	14.65; 11.87	34.16; 12.08
55 hpf (pmole/mg)	44.01; 26.78	90.47; 20.35

This table corresponds to Fig 5Q

Wound healing assay (mean; SD)

Plasmid transfected	EGFP	EGFP-γGH
A375 (healed area, 10^4 pixels)	2.69; 1.12	2.61; 0.8
A549 (healed area, 10^4 pixels)	6.34; 1.88	3.99; 2.52

This table corresponds to Figs 6C and 6D

Single embryonic cell migration assay (mean; SD)

Treatment	E3 buffer (embryo water)		NAC	5-CHO-THF
Plasmid injected	EGFP	EGFP-γGH	EGFP-γGH	EGFP-γGH
Average speed (pixels/frame)	0.87; 0.19	0.5; 0.08	0.77; 0.18	0.85; 0.25
Maximum speed (pixels/frame)	5.99; 0.99	5.63; 1.04	6.69; 1.98	6.4; 1.57
Total distance (pixels)	103.1; 22.66	59.61; 9.03	92.31; 21.27	102.4; 29.47
Maximum distance (pixels)	61.87; 9.89	35.85; 8.11	48.20; 8.24	55.23; 19.96

This table corresponds to Fig 6F-6I

Neural crest cell migration analysis (*sox10* expression) (mean; SD)

Treatment	E3 buffer (embryo water)		NAC
Larvae	CTL	SFD	SFD
% of embryos with delayed migration	13.00; 12.53	52.67; 8.74	8.33; 14.43
Scores of impeded migration	0.44; 0.7	1.43; 0.69	0.58; 0.64

This table corresponds to Fig 6K
